# COVID-19 Pandemic: Age-Related Differences in Measures of Stress, Anxiety and Depression in Canada

**DOI:** 10.3390/ijerph17176366

**Published:** 2020-09-01

**Authors:** Izu Nwachukwu, Nnamdi Nkire, Reham Shalaby, Marianne Hrabok, Wesley Vuong, April Gusnowski, Shireen Surood, Liana Urichuk, Andrew J. Greenshaw, Vincent I.O. Agyapong

**Affiliations:** 1 Cumming School of Medicine, University of Calgary, Calgary AB T2N 1N4, Canada; izu.nwachukwu@ahs.ca (I.N.); marianne.hrabok1@ucalgary.ca (M.H.); 2 Department of Psychiatry, Faculty of Medicine and Dentistry, University of Alberta, Edmonton AB T6G 2R3, Canada; Nnamdi.Nkire@albertahealthservices.ca (N.N.); rshalaby@ualberta.ca (R.S.); liana.urichuk@albertahealthservices.ca (L.U.); andy.greenshaw@ualberta.ca (A.J.G.); 3 Addiction and Mental Health, Alberta Health Services, Edmonton, AB T6G 2R3, Canada; Wesley.Vuong@albertahealthservices.ca (W.V.); April.Gusnowski@albertahealthservices.ca (A.G.); shireen.surood@albertahealthservices.ca (S.S.)

**Keywords:** COVID-19, pandemic, anxiety, depression, stress, e-mental health, age categories, Text4Hope

## Abstract

Background: The spread of COVID-19 along with strict public health measures have resulted in unintended adverse effects, including greater levels of distress, anxiety, and depression. This study examined relative presentations of these psychopathologies in different age groups in a Canadian cohort during the COVID-19 pandemic. Methodology: Participants were subscribers to the Text4Hope program, developed to support Albertans during the COVID-19 pandemic. A survey link was used to gather demographic information and responses on several self-report scales, such as Perceived Stress Scale (PSS), Generalized Anxiety Disorder 7-item (GAD-7) scale, and Patient Health Questionnaire-9 (PHQ-9). Results: There were 8267 individuals who completed the survey, giving a response rate of 19.4%. Overall, 909 (11.0%) respondents identified as ≤25 years, 2939 (35.6%) identified as (26–40) years, 3431 (41.5%) identified as (41–60) years, 762 (9.2%) identified as over 60 years, and 226 (2.7%) did not identify their age. Mean scores on the PSS, GAD-7, and PHQ-9 scales were highest among those aged ≤25 and lowest amongst those aged >60 years old. Conclusions: The finding that the prevalence rates and the mean scores for stress, anxiety, and depression on standardized scales to decrease from younger to older subscribers is an interesting observation with potential implications for planning to meet mental health service needs during COVID-19.

## 1. Introduction

With its discovery in Wuhan, China, and its subsequent rapid spread around the world, the coronavirus disease (COVID-19) pandemic has caused palpable fear [1,2,3] To manage the illness in the absence of a proven cure or an effective vaccine, governments have adopted extreme public health measures including shutting down all but essential services and industries, promoting hand hygiene measures, restricting travel and closing borders, implementing social distancing, self-isolation, and quarantine measures [4]. Typically, social distancing has been achieved through limiting the distance between individuals in public spaces, limiting the number of individuals who are allowed to gather together, self-isolation/quarantine for 14 days after travel or if individuals present with COVID-19 like symptoms or have been in contact with potentially infected individuals. These measures have caused a widespread disruption of both the social fabric of society and economic activities [5]. These abrupt changes to the pattern of human activities have had indirect negative effects on the physical and mental health of individuals across the world.

Self-isolation measures and quarantine, despite their considerable clinical utility, often have unintended adverse effects [6] including greater levels of distress, anxiety, depression, and post-traumatic stress disorder (PTSD). For the current COVID-19 pandemic, published studies examining rates of anxiety and depression are consistently reporting prevalence estimates of around 20% [7,8,9]. A recent meta-analysis reported rates of depression and anxiety that exceed 20% with differences in certain demographic variables such as gender and occupation [10]. In the severe acute respiratory syndrome (SARS) outbreak in Taiwan during late April to mid-May 2003, a relationship between age and the development of psychological symptoms was reported, with younger age groups at higher risk [11]. For the COVID-19 pandemic, several studies have also reported a possible negative relationship between depression, anxiety, PTSD, and age [7,12].

In an online survey of Chinese subjects, prevalence of generalized anxiety disorder and depressive symptoms was significantly higher in participants younger than 35 years than in participants aged 35 years or older [13] with age and amount of time spent focusing on COVID-19 identified as potential risk factors for psychological illness. Individuals ≤35 years of age appear to be more likely to develop anxiety and depressive symptoms during the COVID-19 pandemic [14]. In a nationwide survey examining psychological distress among Chinese people in the COVID-19 pandemic using a COVID-19 Peritraumatic Distress Index (CPDI) [15]; the authors examined frequency of anxiety, depression, specific phobias, cognitive change, avoidance and compulsive behavior, physical symptoms, and loss of social functioning in the past week, with scores on the CPDI ranging from 0 to 100. A CPDI score between 28 and 51 indicates mild to moderate distress while a score ≥52 indicates severe distress. In that study, participants under 18 years had the lowest CPDI scores. Individuals from 18–30 years or >60 years of age presented the highest CPDI scores, thus presenting a more nuanced view of the effect of the pandemic on psychological symptoms across the age spectrum. Possible explanations for their finding include the idea that teenagers and children have shown relatively low morbidity and mortality in the pandemic and therefore may feel less stressed by it and, because of school closures and quarantine measures they may recognize that they have had limited exposure to the coronavirus. Younger adults on the other hand may be exposed to more information about the virus via social media, a factor that has been shown to increase vulnerability [16]. In addition, their loss of social connections with friends may have further increased their vulnerability to mental distress. The highest mortality rates for the virus are reported among the elderly, thus potentially exposing this age group to be more adversely affected psychologically [15]. The majority of initial studies examining the impact of age on stress, anxiety, and depression levels in the current COVID-19 pandemic arise from Asia. This present study sets out to examine the evidence for the impact of age on stress, anxiety, and depression levels in the COVID-19 pandemic from the perspective of a Canadian cohort with the goal of informing policy planning in relation to age-appropriate mental health supports and resource allocations during this COVID-19 pandemic period.

## 2. Methods

This was a cross-sectional survey exploring the mean differences of perceived stress, anxiety, and depression symptom scores among subscribers of various age categories who enrolled in the Text4Hope program. The study recruitment procedures and statistical methods have been described in related papers [17]. In summary, the Text4Hope program is a daily supportive text message service, launched by Alberta Health Services (Alberta, Edmonton), the Provincial Health Authority on 23 March 2020 to support the mental health of Albertans during the COVID-19 pandemic. Subscribers were sent an online survey link with an accompanying message: “To help us evaluate the Text4Hope program’s effectiveness, please complete a short survey….” The survey questions included demographic information such as gender, age, ethnicity, education, relationship status, employment status, and housing status. Respondents also completed clinical self-assessments for stress, anxiety, and depression using the Perceived Stress Scale (PSS), the Generalized Anxiety Disorder 7-item (GAD-7) scale and the Patient Health Questionnaire-9 (PHQ-9), respectively. Participant consent was implied by submission of subscribers’ survey responses. The survey link has no expiry date as enrollment to the Text4Hope program is ongoing. Ethical approval for the research was obtained through the University of Alberta Health Research Ethics Board (Pro00086163).

Data analysis was undertaken using SPSS version 26 (IBM Inc, Endicott, NY, USA) [18]. Demographic characteristics of respondents were summarized in absolute numbers and percentages, by age category. One-way analysis of variance (one-way ANOVA) with two tailed significance (*p*-value < 0.05) was performed to assess the differences between the ethnic groupings and the corresponding mean scores for PSS, GAD-7, and PHQ-9, respectively. As all variables violated the homogeneity of variance assumption based on the Levene Statistic test of homogeneity, we determined statistically significant differences for the mean scores for the various clinical measures across age groups using the Welch F test and a Games–Howell post hoc test.

## 3. Results

Of the 44,992 subscribers who joined Text4Hope in the first 6 weeks, 8267 responded to the online survey invitation, yielding a 19.4% response rate. Our sample size of 8267 indicates that any prevalence rate estimates for the entire sample of 44,992 subscribers would have a 99% confidence interval and a margin of error of only 1.28%. Of the 8267 respondents, 909 (11.0%) identified as ≤25 years, 2939 (35.6%) identified as aged 26–40 years, 3431(41.5%) identified as aged 41–60 years, 762 (9.2%) identified as >60 years, and 226 (2.7%) did not identify their age. The mean age for our sample was 42.09 years (Standard Deviation = 13.44 years).

Additional demographic characteristics of the respondents are shown in Table 1, which indicates a majority of respondents self-identified as female, (*n* = 6991, 87.1%), Caucasian (*n* = 6579, 82.3%), with post-secondary education (*n* = 6835, 85.2%), as employed (*n* = 5883 73.3%), as married, cohabiting, or partnered (*n* = 5706, 71.1%), and as home-owners (*n* = 5194, 65.9%).

The data displayed in Table 2 illustrate the prevalence rates for clinically meaningful stress, anxiety, and depression. These data suggest the prevalence of high/moderate stress, likely GAD and likely MDD were highest in those aged 25 or under and lowest in those aged over 60 years. 

Mean scores for all the respondents were 20.79 (SD = 6.83, *n* = 7589) on the PSS, 9.68 (SD = 5.87, *n* = 6944) on the GAD-7 scale, and 9.43 (SD = 6.29, *n* = 7082) on the PHQ-9 scale.

The data displayed in Table 3 indicate that the mean scores on the PSS, GAD-7, and PHQ-9 scales were highest among those aged 25 years and under and lowest amongst those who were over 60 years old. There is an observed trend for the mean scores for all three scales to decrease with a shift from a younger age bracket to an older age bracket.

Results from the Levene test for homogeneity of variances suggested there was a violation of the assumption of equality of means for the PSS, GAD-7, and PHQ-9 scales (*p* > 0.05). Because of this, it was appropriate to apply the Welch F test and a Games–Howell post hoc test to determine mean score differences on the three scales between the different age groups. Welsh F tests confirmed that the differences between the groups in terms of their mean PSS, GAD-7, and PHQ-9 scores were statistically significant.

There were statistically significant differences between and within age groups for scores on the PSS (F = 319.89, *p* < 0.001), GAD-7 scale (F = 225.23, *p* < 0.001), and PHQ-9 (F = 195.82, *p* < 0.001).

The results of the Games–Howell post hoc test are as presented in Table 4. The results displayed in Table 4 confirm statistically significant differences in mean scores on the PSS, GAD-7, and PHQ-9 scales between each of the age categories and any other age category (*p* < 0.001 for each comparison). The mean scores for the PSS, GAD-7, and PHQ-9 scales declined significantly with a shift from a younger age to an older age group suggesting that older respondents had less stress, anxiety, and depression symptoms compared to younger respondents.

For each of the three scales, the greatest mean differences were observed between respondents who were ≤25 years compared to those >60 years. For the PSS and the GAD-7, the respective mean differences in scores was 8.75, with a 95% CI of 7.89–9.61 and *p* < 0.001, and 6.41, with a 95% CI of 5.57–7.24 and *p* < 0.001. For the PHQ-9, the mean difference in the score between these age groups was 5.88, with a 95% CI of 5.14–6.63 and *p* < 0.001.

Overall, the results suggest a decrease in severity of stress, anxiety, and depression symptoms with increasing age during COVID-19 in a Canadian sample.

## 4. Discussion

Our results indicate that about two-thirds of our respondents were aged between 26 and 60 years, and the remaining respondents were aged either 25 years and under or over 60 years. This contrasts with a study by Gonzalez-Sanguino et al. [7] where the majority of respondents were aged between 18–39 years (56.63%). The respondents in our sample were spread over a wider middle age range. Furthermore, the average age of the sample population in the study by Gonzalez-Sanguino et al. [7] was lower than the average age for our study participants (37.92 vs. 42.09, respectively). Our study results are, however, similar to those of other studies which have a lower representation of the elderly population [7,8]; and this underrepresentation of this important segment of the population may limit us in relation to inferences made for those over 60 years of age in this study.

Our results indicate that the prevalence rates for moderate/high stress, likely GAD, and likely MDD as well as the mean scores on the PSS, GAD-7, and PHQ-9 scales were highest amongst those aged under 25 years, and lowest amongst those over 60 years. This finding is consistent with some previous studies that reported higher scores in stress, anxiety, and depressive symptoms in younger people compared to older ages [7,11,12]. The finding that people aged 60 years and above reported lower scores on our rating scales is both interesting and curious, given that COVID-19 infections have been shown to cause significantly higher morbidity and mortality in this age group compared to the younger age group [19,20]. Since there was stronger emphasis of the need for people over 60 years to take more stringent measures with social distancing and they are also likely to have higher prevalence of underlying medical conditions, it may have been expected that they would be more distressed during the pandemic. On the other hand, older people tend to be less socially mobile than younger ones, thus possibly explaining their reported lower scores on rating scales for stress, anxiety, and depression during a pandemic lockdown. People above 60 years are also more likely to have experienced various major life events in the past, possibly including having lived through past epidemics or pandemics, hence their increased resilience as found in our study. Additionally, younger people, especially those under 25 years, may have perceived their academic, social, occupational, and economic prospects to be more threatened by COVID-19 compared to those over 60 years and this will likely, at least in part, explain their increased stress levels according to our study [13,14].

As outlined in the introduction, several studies have reported lower rates of anxiety and depression in older age groups compared to younger ones [7,11,12]. Another hypothesis that could be propounded to explain this finding include that younger people, especially those under 25 years of age, are known to spend more time on social media and other news outlets. For example, in a 2019 US study, 90% of people aged 18 to 29 were active on social media, compared to 45% of those aged over 65 years [21]. High consumption rates of news about the COVID-19 pandemic have been associated with increased levels of distress [16]. Having said this, one might also have expected that increased opportunities for social connection through social media outlets that are readily available to younger people would limit the impact of physical distancing on them, perhaps compared to older adults.

One group particularly vulnerable to the effects of the COVID-19 pandemic continues to be older adults in senior care homes [19]. At the time this survey was designed in the context of Text4Hope, that fact was not known. It is likely that the majority of seniors responding to the Text4Hope survey are not in care, and this limitation must be taken into account in interpreting these and similar results on decreased severity of mental health indices in the older population. More research directed specifically at understanding the impact of social connectedness with stress, anxiety, and depression is needed to shed more light in this area.

According to the UK Office for National Statistics, a population-based survey found that people over seventy years of age reported feeling happier than those aged 16 to 69 years during the period before a national lockdown was imposed in the wake of COVID-19 in the United Kingdom (UK) [22]. Interestingly, this gap in reported feelings of happiness between the groups decreased by the third week of the lockdown. Again, the UK government recommended stricter social distancing measures for those over 70 years of age, possibly explaining why they would have become increasingly more anxious and distressed as the pandemic continued. In comparison, our study data were collected at a single point at the beginning of the lockdown when social distancing was imposed across the province of Alberta, Canada.

The clinical and practical utility of our study derives mainly from the its potential to serve as a guide to healthcare planners in directing treatment and support services in a more targeted and age-appropriate way during this COVID-19 pandemic and related crisis situations in the future. With the knowledge that younger people, including students [4], tend to suffer disproportionately higher levels of stress, anxiety, and depression, equitable attention must be paid to ensure that their needs are met in all relevant areas. For example, online platforms may be used to deliver psychotherapeutic interventions and support networks to young people in their homes so as to minimize the spread of the virus while mitigating their increased vulnerability to mental distress during the pandemic. Educational institutions and authorities may also need to develop online platforms and portals to aid the delivery of lectures and other learning materials with a view to maintaining as much of their daily structure and routine as possible.

Our study was limited in being a snapshot of self-reported experiences of mental health signs and symptoms at the beginning of the Alberta lockdown, as opposed to a more longitudinal evaluation, especially if administered by a trained clinician. It is possible therefore that data collected a few weeks further down the line from our original data set would reflect similar findings as did the aforementioned UK study [3]. Furthermore, our study is not representative of the population in Alberta either by age or gender [23] and so our findings may not be generalized to the entire population. In addition, although the ANOVA analysis allowed for comparison of the stress, anxiety, and depression levels between all the age groups as a strength, it did not take into account potential confounding factors such as sex, ethnicity, relationship status, employment and education status, which is a limitation. Age is likely to be one of the several factors upon which vulnerability to mental health effects of COVID-19 would be based. In addition, other social determinants of health, along with co-morbid physical health conditions, are known to play significant parts in increasing vulnerability in times of crisis [10]. Any interventions aimed at mitigating mental health effects of COVID-19 must therefore take of all these various factors into account. Finally, our survey did not ask participants about pre-existing stress, anxiety, and depression. It is possible that some respondents had these baseline stress, anxiety, and depression and so the reported scores on the standardized scales may not all be attributable to the COVID-19 pandemic.

## 5. Conclusions

Our results also indicate that both the prevalence rates as well as the mean scores for stress, anxiety, and depression on standardized scales were highest amongst those under 25 years, and lowest amongst those over 60 years. The trend for mean scores across the stress, depression, and anxiety scales to decrease in severity from younger to older age has potential implications for planning to meet mental health service needs during COVID-19. Innovative and cost-effective interventions such as supportive text messaging which are independent of geographic location, are free to the end user, do not require expensive data plans, and can reach thousands of people simultaneously [24,25,26,27,28,29,30,31] could be useful particularly to a younger age population who seem to be most impacted psychologically during the COVID-19 pandemic.

## Figures and Tables

**Table 1:** Age distribution of demographic, clinical, and other characteristics of respondents.

Variables	≤25 Years	26–40 Years	41–60 Years	>60 Years	Overall
	909 (11.0)	2939 (35.6%)	3431 (41.5%)	226 (2.7%)	8267 (100%)
	*n* (%)	*n* (%)	*n* (%)	*n* (%)	*n* (%)
**Gender**					
Male	108 (11.3)	336 (35.0)	396 (41.3)	119 (12.4)	959 (11.9)
Female	776 (11.1)	2568 (36.7)	3009 (43.0)	638 (9.1)	6991 (87.1)
Other	23 (29.1)	33 (41.8)	19 (24.1)	4 (5.1)	79 (1.0)
**Ethnicity**					
Caucasian	646 (9.8)	2355 (35.8)	2905 (44.2)	673 (10.2)	6579 (82.3)
Indigenous	47 (15.9)	124 (42.0)	111 (37.6)	13 (4.4)	295 (3.7)
Asian	88 (22.1)	188 (47.1)	113 (28.3)	10 (2.5)	39 (5.0)
Other	125 (17.2)	261 (36.0)	283 (39.0)	56 (7.7)	725 (9.1)
**Education**					
Less than High School Diploma	199 (62.2)	58 (18.1)	49 (15.3)	14 (4.4)	320 (4.0)
High School Diploma	170 (21.6)	248 (31.5)	276 (35.1)	93 (11.8)	787 (9.8)
Post-Secondary Education	530 (7.8)	2598 (38.0)	3064 (44.8)	643 (9.4)	6835 (85.2)
Other Education	9 (11.1)	30 (37.0)	34 (42.0)	8 (9.9)	81 (1.0)
**Employment Status**					
Employed	358 (6.1)	2407 (40.9)	2832 (48.1)	286 (4.9)	5883 (73.3)
Unemployed	177 (19.1)	349 (37.6)	362 (39.1)	39 (4.2)	927 (11.6)
Retired	0 (0.0)	0 (0.0)	125 (22.9)	420 (77.1)	545 (6.8)
Students	334 (74.6)	95 (21.2)	18 (4.0)	1 (0.2)	448 (5.6)
Other	39 (17.8)	82 (37.4)	84 (38.4)	14 (6.4)	219 (2.7)
**Relationship Status**					
Married/Cohabiting/Partnered	455 (8.0)	2251 (39.4)	2513 (44.0)	487 (8.5)	5706 (71.1)
Separated/Divorced	11 (1.8)	111 (18.3)	383 (63.0)	103 (16.9)	608 (7.6)
Widowed	0 (0.0)	5 (3.9)	49 (38.0)	75 (58.1)	129 (1.6)
Single	426 (28.4)	545 (36.4)	445 (29.7)	83 (5.5)	1499 (18.7)
Other	13 (16.2)	24 (30.0)	31 (38.8)	12 (15.0)	80 (1.0)
**Housing Status**					
Own Home	88 (1.7)	1731 (33.3)	2743 (52.8)	632 (12.2)	5194 (65.9)
Living with Family	513 (65.9)	188 (24.1)	66 (8.5)	12 (1.5)	779 (9.9)
Renting	282 (15.3)	948 (51.5)	530 (28.8)	80 (4.3)	1840 (23.3)
Other	12 (16.9)	25 (35.2)	28 (39.4)	6 (8.5)	71 (0.9)

**Table 2:** Chi-Square test of association between age categories and the prevalence of perceived stress, likely generalized anxiety disorder, and likely major depressive disorder.

Psychological Concern	Age Categories				
	≤25 Years *n* (%)	26–40 Years *n* (%)	41–60 Years *n* (%)	>60 Years *n* (%)	Total Prevalence *n* (%)
**Perceived Stress**					
Moderate or High Stress ^a^	823 (96.3)	2500 (91.1)	2610 (81.9)	475 (68.2)	6408 (85.7)
*Chi ^2^*	353.21				
*p-value*	≤0.001				
Effect Size (Phi)	0.22				
**Generalized Anxiety Disorder (GAD)**					
GAD likely ^b^	515 (65.5)	1481 (58.6)	1090 (37.5)	147 (23.3)	3233 (47.2)
*Chi ^2^*	491.81				
*p-value*	≤0.001				
Effect Size (Phi)	0.27				
**Major Depressive Disorder (MDD)**					
MDD likely ^c^	534 (66.8)	1308 (50.9)	1064 (35.9)	172 (26.4)	3078 (44.1)
*Chi ^2^*	378.10				
*p-value*	≤0.001				
Effect Size (Phi)	0.23				

^a^—Moderate or high stress defined as PSS score ≥ 14; ^b^—Likely GAD defined as GAD-7 scale score ≥ 10; ^c^—Likely MDD defined as PHQ-9 scale score ≥ 10.

**Table 3:** Mean scores on the PSS, GAD-7 Scale and PHQ-9 Scale by age.

Outcome Measures	Age in Years	*n*	Mean	Std. Deviation	Std. Error	95% Confidence Interval for Mean		Minimum	Maximum
						Lower Bound	Upper Bound		
PSS Total Score	≤25	855	25.4	6.29	0.215	24.97	25.82	1	40
	26–40	2743	22	6.209	0.119	21.77	22.23	2	40
	41–60	3185	19.46	6.584	0.117	19.23	19.69	0	40
	>60	696	16.65	6.771	0.257	16.14	17.15	0	39
	Total	7479	20.81	6.833	0.079	20.65	20.96	0	40
GAD-7 Total Score	≤25	786	12.23	5.697	0.203	11.83	12.63	0	21
	26–40	2526	11.12	5.63	0.112	10.9	11.34	0	21
	41–60	2904	8.53	5.625	0.104	8.33	8.74	0	21
	>60	631	6.35	5.172	0.206	5.94	6.75	0	21
	Total	6847	9.71	5.863	0.071	9.57	9.85	0	21
PHQ-9 Total Score	≤25	800	13.05	6.734	0.238	12.59	13.52	0	27
	26–40	2569	10.35	6.086	0.12	10.12	10.59	0	27
	41–60	2961	8.31	5.897	0.108	8.1	8.52	0	27
	>60	652	6.65	5.609	0.22	6.22	7.08	0	27
	Total	6982	9.45	6.29	0.075	9.3	9.6	0	27

**Table 4:** Games-Howell post hoc multiple comparison.

Outcome Measures	(I) Age2 (Years)	(J) Age2 (Years)	Mean Difference (I-J)	Std. Error	Sig.	95% Confidence Interval	
						Lower Bound	Upper Bound
PSS Total Score	≤25	26–40	3.396 *	0.246	0.000	2.76	4.03
		41–60	5.936 *	0.245	0.000	5.31	6.57
		>60	8.747 *	0.335	0.000	7.89	9.61
	26–40	≤25	−3.396 *	0.246	0.000	−4.03	−2.76
		41–60	2.540 *	0.166	0.000	2.11	2.97
		>60	5.351 *	0.283	0.000	4.62	6.08
	41–60	≤25	−5.936 *	0.245	0.000	−6.57	−5.31
		26–40	−2.540 *	0.166	0.000	−2.97	−2.11
		>60	2.811 *	0.282	0.000	2.09	3.54
	>60	≤25	−8.747 *	0.335	0.000	-9.61	−7.89
		26–40	−5.351 *	0.283	0.000	−6.08	−4.62
		41–60	−2.811 *	0.282	0.000	−3.54	−2.09
GAD-7 Total Score	≤25	26–40	1.116 *	0.232	0.000	0.52	1.71
		41–60	3.699 *	0.228	0.000	3.11	4.29
		>60	5.884 *	0.289	0.000	5.14	6.63
	26–40	≤25	−1.116 *	0.232	0.000	−1.71	−0.52
		41–60	2.583 *	0.153	0.000	2.19	2.98
		>60	4.769 *	0.234	0.000	4.17	5.37
	41–60	≤25	−3.699 *	0.228	0.000	−4.29	−3.11
		26–40	−2.583 *	0.153	0.000	−2.98	−2.19
		>60	2.186 *	0.231	0.000	1.59	2.78
	>60	≤25	−5.884 *	0.289	0.000	−6.63	−5.14
		26–40	−4.769 *	0.234	0.000	−5.37	−4.17
		41–60	−2.186 *	0.231	0.000	−2.78	−1.59
PHQ-9 Total Score	≤25	26–40	2.701 *	0.267	0.000	2.02	3.39
		41–60	4.744 *	0.262	0.000	4.07	5.42
		>60	6.405 *	0.324	0.000	5.57	7.24
	26–40	≤25	−2.701 *	0.267	0.000	−3.39	−2.02
		41–60	2.043 *	0.162	0.000	1.63	2.46
		>60	3.704 *	0.250	0.000	3.06	4.35
	41–60	≤25	−4.744 *	0.262	0.000	−5.42	−4.07
		26–40	−2.043 *	0.162	0.000	−2.46	−1.63
		>60	1.661 *	0.245	0.000	1.03	2.29
	>60	≤25	−6.405 *	0.324	0.000	−7.24	−5.57
		26–40	−3.704 *	0.250	0.000	−4.35	−3.06
		41–60	−1.661 *	0.245	0.000	−2.29	−1.03

* The mean difference is significant at the 0.05 level.
